# Choroidal vascularity index in thyroid-associated ophthalmopathy: a cross-sectional study

**DOI:** 10.1186/s40662-021-00242-6

**Published:** 2021-04-30

**Authors:** Pasquale Loiudice, Marco Pellegrini, Michele Marinò, Barbara Mazzi, Ilaria Ionni, Giuseppe Covello, Michele Figus, Marco Nardi, Giamberto Casini

**Affiliations:** 1 Department of Surgical, Ophthalmology Unit, Medical, Molecular and Critical Area Pathology, University of Pisa, Via Savi, 10, 56126 Pisa, Italy; 2 Ophthalmology Unit, S.Orsola-Malpighi University Hospital, University of Bologna, Bologna, Italy; 3 Department of Clinical and Experimental Medicine, Endocrinology Unit I, University Hospital of Pisa, Pisa, Italy

**Keywords:** Choroidal vascularity index, Choroidal vasculature, Enhanced depth optical coherence tomography, Image binarization, Luminal area, Subfoveal choroidal thickness, Thyroid-associate ophthalmopathy

## Abstract

**Background:**

Hemodynamic changes have been observed in patients with Graves’ disease. The aim of our study was to evaluate choroidal vascular change using the choroidal vascularity index (CVI) in patients with thyroid-associated ophthalmopathy (TAO).

**Methods:**

In this cross-sectional observational study, 40 patients affected by TAO were recruited. Forty healthy individuals, matched for age and sex, served as controls. Foveal enhanced-depth imaging optical coherence tomography scans were obtained from all participants. Images were binarized using the ImageJ software and luminal area (LA) and total choroidal area (TCA) were measured. CVI was calculated as the proportion of LA to TCA. The relation between CVI or subfoveal choroidal thickness (SFCT) and clinical activity score, exophthalmometric value, diplopia status, gender, and age was evaluated.

**Results:**

CVI was significantly higher in patients with TAO (*P* = 0.004). No significant difference was observed in SFCT (*P* = 0.200) and TCA (*P* = 0.153) comparing TAO patients and healthy controls. LA was significantly higher in TAO group (*P* = 0.045). On multiple regression analysis, CVI was associated with TCA (*P =* 0.043). No association was found between SFCT or CVI and TCA, clinical activity score, exophthalmometric value, Inami value, diplopia status, gender or age (*P* > 0.05).

**Conclusions:**

This is the first study that has demonstrated an increase in CVI in eyes with TAO compared with healthy controls and has assessed its association with clinical features.

## Background

Thyroid-associated ophthalmopathy (TAO), also called Graves’ ophthalmopathy or Graves’ orbitopathy, is an autoimmune disorder involving the orbital tissue, commonly found in patients with Graves’ disease [1]. Symptomatology ranges from ocular irritation and dryness in mild forms to redness, chemosis, edema and erythema of eyelids and diplopia [1]. Potential sight-threatening conditions such as corneal ulceration and compressive optic neuropathy may manifest in the most severe cases [2].

The pathogenesis of TAO has not been completely elucidated and is considered as the result of a combination of genetic and environmental factors [3]. Several risk factors have been investigated, including tobacco smoking and number of cigarettes smoked per day, older age at diagnosis of Graves’ hyperthyroidism, longer duration of the disease, uncontrolled thyroid dysfunction and prior radioactive iodine treatment [4–7]. TAO is more common in women but is more severe in men [8]. Recent evidence indicates a possible role of orbital fibroblasts in the pathogenesis of TAO even though the reason for anatomic site-specific localization remains uncertain. Once activated by thyroid-stimulating immunoglobulins, fibroblasts proliferate and produce pro-inflammatory cytokines and extracellular matrix constituents [9]; this results in hygroscopic swelling of extraocular muscles and expansion of the adipose tissue. Three pathophysiological steps have been identified in the so-called “Cone model”: (a) expansion of rectus muscles and fat, forward displacement of extra-conal fat; (b) axial advancement of the globe and rectus muscle stretching; (c) impaired posterior venous drainage and reversal of conjunctival venous flow with eyelid edema [10].

Hyperthyroidism may also induce an increase in heart rate, cardiac output, and systolic blood pressure [11, 12]. In this setting, hemodynamic changes have been observed in many organs, including the eyes [13, 14]. A reduction in pulsatile ocular blood flow, pulse amplitude and pulse volume [15] and an increase in retinal blood flow have been observed in patients with Graves’ disease [16].

As the choroid is the main vascular layer of the eye, several studies investigated choroidal thickness (CT) changes in patients with TAO [17–21]. However, discrepancy in clinical findings and the clinical activity of the disease has been observed in various studies [17–21].

Recently, a new optical coherence tomography (OCT) parameter termed choroidal vascularity index (CVI) has been introduced to investigate the choroidal vasculature [22, 23]. It was obtained by binarization of optical coherence tomography images and was defined as the proportion of luminal area (LA) to total cross-sectional choroidal area (TCA) [24]. CVI gained increasing interest since it was observed to not be influenced by age, gender, refractive error, axial length or intraocular pressure (IOP) [24].

Therefore, our study aimed to assess changes in the choroidal vasculature using the CVI in eyes with TAO, and to compare the results with age- and sex-matched healthy controls. An additional objective was to evaluate the relation between CVI and clinical activity score, exophthalmometric value, diplopia status, gender and age.

## Methods

In this cross-sectional single center study, we included 40 patients affected by TAO referred to Ophthalmology Unit by the Endocrinology Department of Pisa University Hospital. Forty healthy individuals, matched for age and sex, served as controls. This study received approval by the local Institutional Review Board (Comitato Etico, Area Vasta Nordovest, register number 18781) and was conducted in adherence to the tenets of the current version of the Declaration of Helsinki (64th WMA General Assembly, Fortaleza, Brazil, October 2013). All patients signed an informed consent form.

All subjects underwent a complete ophthalmological examination. The following data were collected: age, gender, visual acuity, intraocular pressure (IOP) (Goldmann applanation tonometry), biomicroscopy findings, clinical activity score (CAS), concomitant and previous therapy, exophthalmometric values and motility status. Ocular proptosis was measured by a Hertel exophthalmometer. The items included in the CAS grading scale were: (a) spontaneous retrobulbar pain; (b) pain on attempted upward or downward gaze; (c) eyelid erythema; (d) eyelid edema; (e) conjunctival hyperemia; (f) conjunctival chemosis; (g) inflammation of caruncle or plica. Assigning 1 point for each item, TAO was classified as active if CAS was ≥ 3 [25]. The presence of subjective diplopia in primary gaze position was graduated using Gorman score (0: no diplopia, 1: intermittent diplopia, 2: inconstant diplopia, and 3: constant diplopia) [26]. Inclusion criteria were diagnosis of Graves’ disease in the last 12 months; first episode of TAO; age between 25 and 45 years; euthyroidism in treatment with anti-thyroid drugs; refractive value (spherical equivalent) within the range − 3 diopters (D) to + 3 D. We excluded patients with a history of radiotherapy or thyroidectomy; previous treatment with corticosteroids in the 3 months before enrolment; any ocular or systemic disease that could interfere with the measurements of this study such as glaucoma, diabetic retinopathy, hypertensive retinopathy, previous vitreoretinal surgery, and retinal vein occlusion. The control group comprised of 40 healthy subjects with the same age range (25–45 years) and the same male: female proportion (4: 1) vs. the study group. They had no history of ocular or systemic disease and minimal refractive error (spherical equivalent) of ± 3 D.

Spectral-domain optical coherence tomography (SD-OCT, Spectralis; Heidelberg Engineering, Germany, Software version 6.9) was performed in all subjects using the enhanced-deep image (EDI) mode to obtain a better visualization of the choroid. A volume scan of 20° × 20° centered on the fovea was obtained for each eye. A single B-scan was an average of 20 frames and 240 μm apart from the next B-scan.

Subfoveal choroidal thickness (SFCT) was manually measured using the caliper tool embedded in the software of the instrument. Two trained masked examiners independently analyzed all the OCT scans and manually measured the SFCT, identified the choroidal boundaries and processed the images for binarization. The average of the measurements of the two examiners was considered for statistical analysis. Acquisitions were performed at the same time of the day (12: 00–14: 00) to avoid diurnal variations. Images quality was checked just after acquisition and immediately repeated if necessary. Only scans with at least signal strength ≥ 6 and clearly identifiable choroid-scleral junction were taken for further analysis.

### Binarization of images

**Fig. 1:**
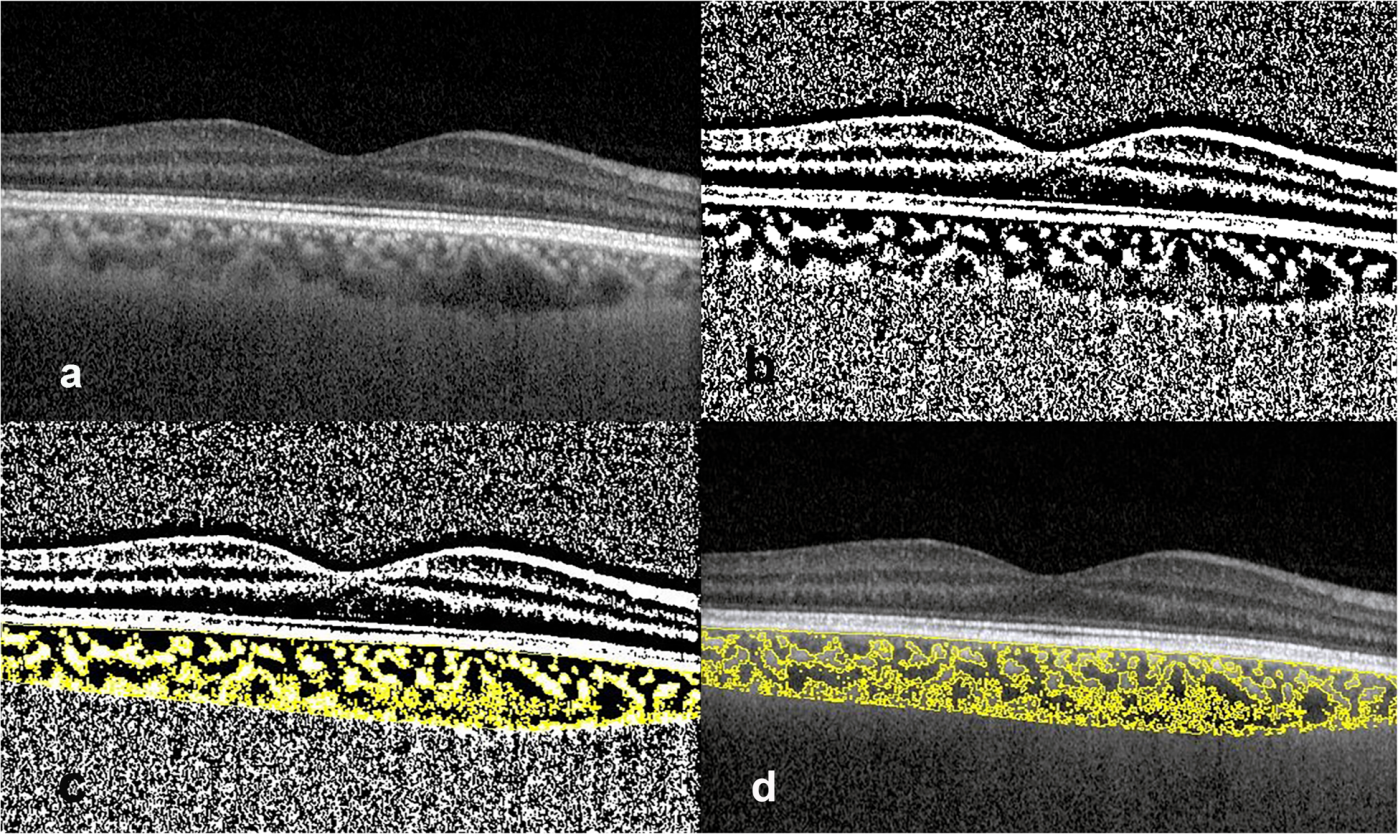
Binarization and identification of the luminal and stromal areas of the choroid. Spectral-domain optical coherence tomography (SD-OCT) acquired using enhanced-depth image (EDI) mode. **a** Original subfoveal scan; **b** The image was downgraded to 8-bit and Niblack auto local threshold was applied; **c** Color threshold was used to select luminal area; **d** Overlay of the region of interest on the original image

The same foveal scan used for CT measurement was processed using the open-source software ImageJ (version 1.52; National Institutes of Health, USA, http://imagej.nih.gov/ij). The polygon tool was used to select the TCA. The selection was added to the region of interest (ROI) manager. The image was then downgraded to 8-bit and adjusted with Niblack auto local threshold. Color threshold was used to select the LA which was added to the ROI manager. CVI was calculated as the proportion of LA to TCA. Stromal area (SA) was calculated by subtracting LA from TCA (Fig. 1).

### Statistical analysis

Statistical analysis was performed using the SPSS software version 20.0 for Windows (SPSS Inc., Chicago, IL, USA). Continuous variables were expressed as mean ± standard deviation, and quantitative variables were expressed as frequency (%). The normality of distribution of data was assessed using Kolmogorov-Smirnov and Shapiro-Wilk tests. Intraclass Correlation Coefficient (ICC) was calculated to evaluate the correlation between right and left eyes. Differences in SFCT, TCA, SA, LA and CVI were assessed applying a two-side independent sample *t*-test*.* Univariate linear regression analysis was performed indicating CVI and SFCT as dependent variables and TCA, CAS, exophthalmometric value, Inami value, diplopia grade, gender and age as independent variables. Covariates with a *P*-value < 0.2 in univariate analyses were included in the multivariable analysis. *P* values < 0.05 were considered statistically significant; 95% confidence intervals (CI) were presented.

## Results

Both eyes of 80 patients, 21 males and 59 females, were included in this study. Forty cases were diagnosed with TAO and 40 subjects served as controls. Mean age was 39.30 ± 4.54 years (range 30–45 years) and 37.45 ± 4.44 years (range 28–45 years) respectively; the difference was not significant (*P* = 0.069, *t*-test). Since there was a strong correlation between right and left eye variables, only data of the right eyes were included in the statistical analysis. Indeed, the ICC value for SFCT was 0.947 (95% CI 0.899–0.972, *P* < 0.001) and for CVI was 0.953 (95% CI 0.906–0.974, *P* < 0.001).

Mean values of TCA, LA, SA, CVI and SFCT are displayed in Table 1.

**Table 1:** Choroidal parameters in patients with thyroid-associated ophthalmopathy and healthy controls

Parameter	TAO group	Control group	*P*
Subfoveal choroidal thickness (μm)	308.08 ± 73.37	288.90 ± 58.32	0.200
Total choroidal area (mm^2^)	0.61 ± 0.21	0.55 ± 0.11	0.153
Luminal area (mm^2^)	0.39 ± 0.14	0.34 ± 0.72	0.045
Stromal area (mm^2^)	0.21 ± 0.06	0.21 ± 0.05	0.927
Choroidal vascularity index (%)	64.78 ± 3.28	62.19 ± 4.44	0.004

*TAO* = thyroid-associated ophthalmopathy

No significant differences were observed in SFCT, TCA and SA comparing patients with TAO and healthy controls (all *P* > 0.05). LA was significantly higher in TAO subjects when compared with controls (*P* = 0.045).

Mean CVI significantly differed between TAO patients (64.78 ± 3.28%, range: 52.60–72.13%) and healthy controls (62.19 ± 4.44%, range: 51.89–70.23%) (*P* = 0.004, *t-*test). We performed a subgroup analysis considering only patients with CAS ≥ 3 (28 subjects, 23 females). No significant differences were observed between patients with active TAO and controls in TCA (0.58 ± 0.18 mm^2^ and 0.53 ± 0.10 mm^2^, respectively), SA (0.207 ± 0.055 mm^2^ and 0.208 ± 0.054 mm^2^, respectively) and SFCT (316.68 ± 74.94 mm^2^ and 281.93 ± 49.75 mm^2^, respectively) (all *P* > 0.05). LA (0.38 ± 0.13 mm^2^ and 0.32 ± 0.06 mm^2^, respectively, *P* = 0.046) and CVI (64.37 ± 3.34% and 61.42 ± 4.90%, respectively, *P* = 0.011) were significantly higher in patients with active TAO. Mean exophthalmometric value in TAO patients was 22.13 ± 2.86 (95% CI 21.29–23.13); mean CAS was 3.48 ± 1.78, (95% CI 2.90–4.05); mean Inami value was 107.03 ± 4.25, (95% CI 105.66–108.39). Active TAO was present in 28/40 patients at the time of the ocular examination. Diplopia was absent in 15 subjects, intermittent in 10, inconstant in 10 and constant in 5. Univariate linear regression analysis was performed for age, gender, presence or absence of diplopia, CAS, degree of exophthalmos, Inami, TCA, LA and SA and their impact on SFCT and CVI. In the univariate analysis, CVI was associated with TCA, LA and SFCT *P* = 0.010; *P* = 0.001 and *P* = 0.043, respectively). In the multivariable analysis, CVI was associated with TCA and LA (all *P <* 0.001) (Table 2).

**Table 2:** Associations between choroidal thickness and choroidal vascularity index and clinical parameters in patients with thyroid-associated ophthalmopathy

	Univariate analysis			Multivariate analysis		
Parameter	β	95% CI	*P*	β	95% CI	*P*
SFCT						
Age	−0.017	−5.58 – 5.03	0.917		–	
Gender	−0.018	−60.17 – 53.77	0.910		–	
Diplopia (yes vs. no)	0.071	−38.45 – 59.60	0.665		–	
CAS	0.281	−1.389 – 24.55	0.079	0.266	−1.99 – 23.89	0.095
Exophthalmometry	−0.011	−8.69 – 8.11	0.944		–	
Inami	−0.167	−8.46 – 2.69	0.302		–	
TCA	−0.183	0.00–0.00	0.258		–	
LA	−0.211	0.00–0.00	0.191	−0.190	0.00–0.00	0.229
SA	−0.108	0.00–0.00	0.506		–	
CVI						
Age	0.060	−0.19 – 0.28	0.713		–	
Gender	0.023	−2.37 – 2.72	0.889		–	
Diplopia *(yes* vs. *no)*	0.016	−2.09 – 2.30	0.924		–	
CAS	−0.231	−1.01 – 0.16	0.152	0.016	−0.13 – 0.19	0.699
Exophthalmometry	−0.095	−0.48 – 0.26	0.559		–	
Inami	0.206	−0.09 – 0.41	0.202		–	
TCA	0.402	0.00–0.00	**0.010**	−6.307	0.00–0.00	**< 0.001**
LA	0.513	0.00–0.00	**0.001**	6.757	0.00–0.00	**< 0.001**
SA	0.123	0.00–0.00	0.450		–	
SFCT	−0.322	−0.02 – 0.00	**0.043**	−0.057	−0.006 – 0.001	0.189

*SFCT* = subfoveal choroidal thickness; *CAS* = clinical activity score; *TCA* = total choroidal area; *LA* = lumen area; *SA* = stromal area; *CVI* = choroidal vascularity index; *CI* = confidence interval, significant *P* values are in bold

## Discussion

Located between the retina and sclera, the choroid is the main vascular layer of the eye. It provides oxygen and nourishment to the fovea and the outer layers of the retina. It is composed of three different zones: the choriocapillaris, the Sattler’s layer with medium size vessels and the Haller’s layer, adjacent to the scleral boundary, with large vessels [27]. The choroid is supplied by posterior ciliary arteries originating from the ophthalmic artery that derives from the internal carotid artery [28]. Reflux blood is collected by the vortex veins, tributary of the ophthalmic vein. Due to the valve-less structure of the ophthalmic veins, choroidal vasculature may be influenced by systemic conditions that have an impact on venous blood flow [29].

Several methods have been proposed to investigate choroidal changes in many pathological conditions, such as histopathological assessment, pulsatile blood flow tonometry, doppler flowmetry, wavelet augmented ultrasound, fluorescein and indocyanine green angiography [30–32]. However, their applicability in daily practice and research settings was limited by the lack of reliability and repeatability or inadequate quantitative parameters for analysis [30].

.Thanks to technological advances, OCT has gained a leading role as it allows us to obtain high-resolution in vivo images in a fast and non-invasive way. In EDI mode acquisition, the lens of the instrument is moved closer to the eye and the zero-delay line is settled beside the choroid; this improves the visualization of the choroid and inner sclera [33]. SFCT measurements obtained with EDI OCT have shown a high intra- and inter-observer reproducibility [34].

Starting from the segmentation method proposed by Sonoda and colleagues [35, 36], Agrawal and co-workers proposed the introduction of a novel OCT marker called CVI, defined as the proportion of LA to TCA [24]. CVI was analyzed in several studies in normal and pathologic conditions including age-related macular degeneration, central serous chorioretinopathy, open-angle glaucoma, Type 2 diabetes and Vogt-Koyanagi-Harada disease [37–41].

In this study, we found that CVI was higher in eyes with TAO than in healthy controls, despite similar SFCT. Increased CT was reported in eyes with TAO by different studies in the last 4 years, summarized in Table 3 [17–21].

**Table 3:** Synthesis of previously reported findings regarding choroidal thickness in patients with Graves’ disease

Authors	Methods	Nationality of the study population	Results	Correlation with ocular parameters	Correlation with systemic parameters	Study limitations
Cagiltay and Akay 2018 [9]	Subfoveal CT; at 500 μm, 1000 μm, and 1500 μm temporal and nasal to the fovea	Turkish	Increased subfoveal, mean and temporal CT; no difference in nasal CT	Correlation with VISA score No correlation with exophthalmometry, axial length	No correlation with disease duration, mean blood pressure	Superior or inferior CT not measured
Bruscolini et al.2018 [10]	Subfoveal CT	Italian	Increased subfoveal CT	Correlation with CAS and exophthalmometry	No correlation with disease duration	Small sample size (*n* = 18); lack of perifoveal measurements
Özkan et al.2016 [11]	Subfoveal CT	Turkish	Increased subfoveal CT	Correlation with CAS and elongated VEP P100	N/A	Only SFCT was examined; small sample size
Lai et al. 2019 [12]	Subfoveal CT; at 1 mm and 2 mm nasal, temporal, inferior and superior to the fovea; peripapillary region	Chinese	Increased CT in all point except at 2 mm inferior to the fovea and at peripapillary region	Association with axial length, exophthalmometry and BCVA No association with IOP and CAS	No association with age, gender, duration of TAO, history of smoking	Most patients with CAS < 4
Yu and Zhang 2018 [13]	Subfoveal CT; at 1500 μm and 3000 μm nasal and temporal to the fovea	Chinese	CT increased in all points	No relationship with CAS, degree of exophthalmos	No relationship with T3, T4, TSH, TRAb levels	Lack of superior and inferior measurements
Çalışkan et al. 2017 [14]	Subfoveal CT, at 1.5-mm and 3.0-mm nasal and temporal to the fovea	Turkish	Increased CT	Correlation with CAS, IOP	Correlation with age, disease activity	Wide range of patient age (21–65 years)

*VISA* = vision, inflammation, strabismus, and appearance; *CT* = choroidal thickness; *CAS* = clinical activity score; *VEP* = visual-evoked potential; *N/A* = not appliable; *SFCT* = subfoveal choroidal thickness; *BCVA* = best-corrected visual acuity; *IOP* = intraocular pressure; *T3* = triiodothyronine; *T4* = tetraiodothyronine; *TRAb* = thyroid-stimulating hormone receptor antibody; *TSH* = thyroid-stimulating hormone

CT was measured in the subfoveal region and at different distances from the fovea. In our study, no significant difference in SFCT was found comparing TAO patients and age- and sex-matched healthy controls, though a statistical trend (*P =* 0.055) was found in the subgroup analysis considering only patients with CAS ≥ 3. However, the smaller sample size may limit the statistical power. A possible explanation may be found in the heterogenicity of the study population since most of the studies reported a wider age range (20–70 years) compared with our subjects (25–45 years). A negative correlation has been reported between CT and age, with approximately a mean CT decrease of 1.5 μm for each year’s increase in age [42]. Eyes of younger individuals may better compensate for modification in choroidal blood flow due to systemic factors and inflammation. Furthermore, in long-lasting disease, the chronic inflammatory insult may result in choroidal vasculopathy and atrophic involution [43]. Recently, Del Noce et al. observed differences in choroidal vascular blood flow in patients with TAO using Angio-OCT when compared with healthy controls [44]. The use of CT as a biomarker has some intrinsic limitations including its circadian fluctuations and its dependence on gender, age and refractive status. Contrarily, current research indicates that CVI is less influenced by physiologic parameters and has minor variability than CT [23].

The choroid is composed of blood vessels surrounded by extracellular matrix. Modifications in CT did not indicate which component was more affected and in what proportion. Furthermore, an increase in one component may be compensated by a reduction of the other. For these reasons, CT is only unfairly representative of the complete choroidal structural modifications.

CVI is the proportion of choroid vasculature to overall choroidal area. An increase in CVI may depend on either an increase in the diameter of the choroidal blood vessels or in the number of blood vessels within a selected region. An increase in retinal blood flow has been observed in active TAO patients [13, 15]. Furthermore, Graves’ orbitopathy is characterized by swelling of extraocular muscles and orbital tissue and fat. The expansion of intraorbital contents in TAO patients is hindered by the inextensible rigid bony walls of the orbit. Consequently, the eyeball is compressed by the expansion of fat and muscles; this may have implications for IOP levels and venous drainage. Increased values of IOP have been documented in patients with TAO when comparing with healthy controls [45]. Reverse blood flow of the superior ophthalmic vein and venous stasis in the orbit have been observed in patients with TAO [46–48]. CVI was found to be higher in other clinical conditions characterized by increased venous pressure such as in patients with carotid-cavernous fistula [49].

.In this study, we did not find any correlation between CVI or SFCT and disease-related ocular parameters such as CAS and exophthalmometric values. This is in contrast with the results of Bruscolini and colleagues [18], Çalışkan and colleagues [50] and Özkan and colleagues [19], but is consistent with the findings of Yu and Zhang [21], and partially concords with Lai and co-workers [20] and Cagiltay and co-workers [17]. CT appeared to not be correlated with systemic parameters including disease duration, mean blood pressure, age, gender and thyroid hormone levels.

Exophthalmometric values of healthy subjects vary among populations depending on ethnicity, ranging from 16.5 mm in White men to 18.5 mm in Black men. Values in women are lower: 15.4 mm and 17.8 mm, respectively [51]. Asians have lower exophthalmometric values than Caucasians [52, 53]. partially imputable to a tighter orbital septum limiting forward movement of the globe [54]. Exophthalmometric values of TAO patients were higher than in the general population as a result of the propulsive forces that occur in the orbit of TAO patients.

A limitation of this study is that the identification of the choroidal boundaries and the measurement of CT are subjective and operator-dependent processes. Furthermore, measurement of CVI was performed in a 2D scan image across the fovea. A volume scan covering the entire macula might provide more information and reduce sampling error.

## Conclusions

CVI was found to be increased in eyes of subjects with TAO. CVI was significantly associated with TCA, LA and SFCT in univariate analysis and with TCA and LA in multivariable linear regression analysis. No association was found between either CVI or SFCT and age, gender, presence of diplopia, CAS, exophthalmometry and Inami. This is the first study that has compared CVI in eyes with TAO and healthy controls and has assessed its association with clinical features; it can, therefore, serve as a starting point for further prospective research.

## Data Availability

The datasets used and/or analyzed during the current study are available from the corresponding author on reasonable request.

## Abbreviations

CAS: Clinical activity score
CI: Confidence interval
CT: Choroidal thickness
CVI: Choroidal vascularity index
EDI: Enhanced deep image
ICC: Intraclass correlation coefficient
IOP: Intraocular pressure
LA: Luminal area
ROI: Region of interest
SA: Stromal area
SD-OCT: Spectral domain – optical coherence tomography
SFCT: Subfoveal choroidal thickness
TAO: Thyroid-associate ophthalmopathy
TCA: Total choroidal area
